# The Regulatory Role of H19/miR-181a/ATG5 Signaling in Perinatal Nicotine Exposure-Induced Development of Neonatal Brain Hypoxic-Ischemic Sensitive Phenotype

**DOI:** 10.3390/ijms23136885

**Published:** 2022-06-21

**Authors:** Yong Li, Yanyan Zhang, Andrew Walayat, Yingjie Fu, Bailin Liu, Lubo Zhang, Daliao Xiao

**Affiliations:** Lawrence D. Longo MD Center for Perinatal Biology, Division of Pharmacology, Department of Basic Sciences, Loma Linda University School of Medicine, Loma Linda, CA 92350, USA; yoli@llu.edu (Y.L.); yanyanzhang@llu.edu (Y.Z.); awalayat@students.llu.edu (A.W.); yifu@llu.edu (Y.F.); bailinliu@llu.edu (B.L.); lzhang@llu.edu (L.Z.)

**Keywords:** nicotine, neonatal HIE, miR-181a, ATG5, lncRNA H19

## Abstract

Nicotine exposure either from maternal cigarette smoking or e-cigarette vaping is one of the most common risk factors for neurodevelopmental disease in offspring. Previous studies revealed that perinatal nicotine exposure programs a sensitive phenotype to neonatal hypoxic-ischemic encephalopathy (HIE) in postnatal life, yet the underlying mechanisms remain undetermined. The goal of the present study was to determine the regulatory role of H19/miR-181a/ATG5 signaling in perinatal nicotine exposure-induced development of neonatal brain hypoxic-ischemic sensitive phenotype. Nicotine was administered to pregnant rats via subcutaneous osmotic minipumps. All experiments were conducted in offspring pups at postnatal day 9 (P9). Perinatal nicotine exposure significantly enhanced expression of miR-181a but attenuated autophagy-related protein 5 (ATG5) mRNA and protein levels in neonatal brains. Of interest, miR-181a mimicking administration in the absence of nicotine exposure also produced dose-dependent increased hypoxia/ischemia (H/I)-induced brain injury associated with a decreased ATG5 expression, closely resembling perinatal nicotine exposure-mediated effects. Locked nucleic acid (LNA)-miR-181a antisense reversed perinatal nicotine-mediated increase in H/I-induced brain injury and normalized aberrant ATG5 expression. In addition, nicotine exposure attenuated a long non-coding RNA (lncRNA) H19 expression level. Knockdown of H19 via siRNA increased the miR-181a level and enhanced H/I-induced neonatal brain injury. In conclusion, the present findings provide a novel mechanism that aberrant alteration of the H19/miR-181a/AGT5 axis plays a vital role in perinatal nicotine exposure-mediated ischemia-sensitive phenotype in offspring and suggests promising molecular targets for intervention and rescuing nicotine-induced adverse programming effects in offspring.

## 1. Introduction

Maternal smoking is one of the most common modifiable perinatal insults which are closely correlated with an increased risk of developmental disease including hypoxic-ischemic encephalopathy (HIE) in offspring [1,2,3,4,5,6]. HIE is one of the leading causes of neonatal morbidity (25%) and mortality (15–20%) globally, with an incidence of 1 to 6 per 1000 term newborns and presents with diverse severe long-lasting neuropsychiatric deficits, including cerebral palsy, seizure, and cognitive retardation in infants and children [7,8,9]. Despite continued progress in perinatal medicine in recent years, currently universally accepted definitive interventions for HIE are still lacking, other than the therapeutic hypothermia applied in limited clinical settings [10,11]. Our previous studies have revealed that perinatal nicotine exposure impairs fetal brain development and induces a DNA hypermethylation at the gene promoter region of angiotensin II receptor (AT2R), leading to AT2R gene repression and consequently developing a hypoxic-ischemic sensitive phenotype in postnatal life [12,13]. However, the underlying molecular epigenetic mechanisms such as the regulatory role of non-coding RNAs in perinatal nicotine exposure-induced development of a hypoxic-ischemic sensitive phenotype remain undetermined. Thus, it is warranted to further investigate its underlying pathological mechanisms and identify potential molecular therapeutic targets for the prevention and effective intervention of neonatal HIE.

It is well recognized that ischemic brain insult is a complicated process, which implicated diverse brain pathologies. Large amounts of evidence have indicated the pivotal roles of miRNAs in the brain development and various brain pathologies [14,15,16,17]. Specifically, growing studies demonstrated that miR-181a plays an important role in the pathogenesis of ischemic brain insults [18,19,20,21,22]. However, the majority of such studies were conducted on mature, fully developed animal brains. Recent evidence reported that cigarette smoking and nicotine exposure can significantly alter the miRNAs profiles in both human and animal models [23,24]. In addition, previous studies have also shown that nicotine can selectively affect specific miRNA expression level [25,26,27]. It is reported that maternal nicotine exposure adversely affects fetal hemodynamics, leading to intrauterine growth restriction (IUGR) and adverse cardiac development in both fetuses and newborns, inducing diverse congenital heart defects such as atrial and ventricular septal defects, hypoplastic left ventricle, thickened aortic and pulmonary valves, and ventricular hypertrophy, and reduced coronary artery size and vessel abundance, which may contribute to an increased risk of stillbirth and sudden infant death syndrome in offspring [28,29]. One of our recent studies showed that perinatal nicotine exposure selectively altered the vascular miR-181a level, which directly regulated coronary vascular tone, and contributed to enhanced heart dysfunction and heart ischemia/reperfusion (I/R) injury in offspring [30]. These findings suggest that miR-181a-mediated signaling may be a crucial epigenetic mechanism underlying perinatal nicotine exposure-induced brain ischemia-sensitive phenotype, which could be considered as a novel therapeutic molecular target. Of interest, recent studies documented that the long non-coding RNA (lncRNA) H19, a highly abundant and conserved imprinted gene, is implicated in many essential biological processes and diverse cardiovascular pathologies [31]. Emerging evidence has also demonstrated an important role of lncRNA H19 in brain pathology and its interaction with miR-181a [32,33,34,35]. Specifically, accumulating studies revealed the vital roles of lncRNA H19 in cell cycle regulation, including various neural stem cells (NSCs). It was documented that lncRNA H19 promotes NSC proliferation, self-renewal and differentiation, contributing to endogenous neurogenesis and improvement of neurological function during stroke recovery [36]. In addition, Wang et al. also revealed that lncRNA H19 overexpression relieves hypoxia-induced NSCs injury by downregulating miR-107 expression, implying close interactions between lncRNA H19 and miRNAs [37]. However, the functional roles of lncRNA H19 in the developing brain and its interactions with miR181a in the setting of nicotine exposure-mediated HIE remain unknown.

The aim of the present study was to determine the regulatory role of H19/miR-181a/ATG5 signaling in perinatal nicotine exposure-induced development of neonatal brain hypoxic-ischemic sensitive phenotype. Our hypothesis is that perinatal nicotine exposure attenuated lncRNA H19 expression but enhanced miR-181a expression, which leads to ATG5 repression and consequent development of a brain hypoxic-ischemic sensitive phenotype in postnatal life. To test our hypothesis, we first examined the effect of perinatal nicotine exposure on miR-181a expression and autophagy-related gene (ATG5) expression levels in the neonatal rat brain. Then, we determined if activation of miR-181a signaling plays a key role in the pathogenesis of neonatal HIE, and further confirmed that inhibition of miR-181a signaling rescued the nicotine exposure-induced brain sensitive phenotype to neonatal HIE. In addition, we also determined that lncRNA H19 as an upstream signaling negatively regulated miR-181a expression in neonatal rat brains. Our current study provides novel evidence of the pivotal roles of miR-181a in the pathogenesis of HIE and confers a new insight of molecular epigenetic mechanism linking maternal cigarette smoking with the heightened neonatal HIE vulnerability in offspring.

## 2. Results

### 2.1. Perinatal Nicotine Exposure Enhanced Expression of miR-181a and Attenuated the Levels of Both ATG5 mRNA and Protein in Neonatal Rat Brain

**Figure 1:**
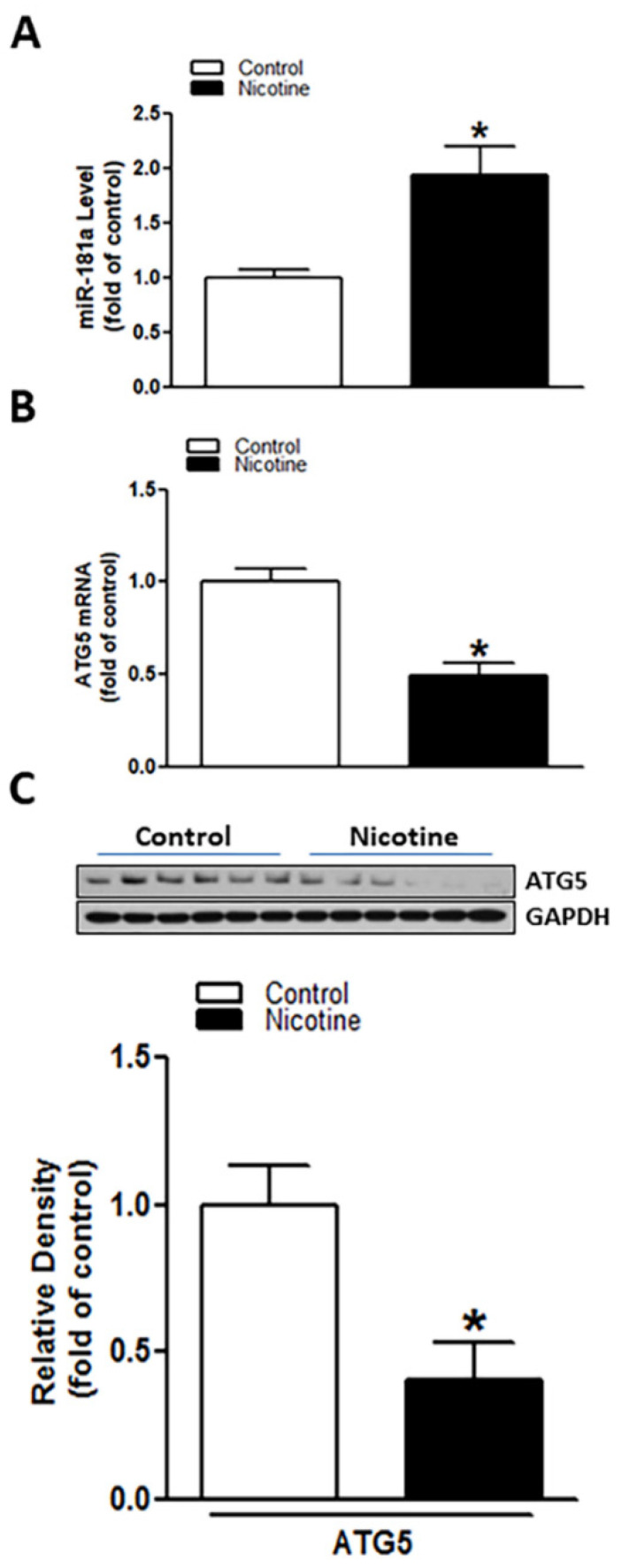
Perinatal nicotine exposure induced miR-181a over-expression and autophagy-related gene 5 (ATG5) repression in neonatal rat brain. Brains were isolated from 9-day-old neonatal rat pups in both saline control and nicotine exposed groups. qRT-PCR analysis was employed to evaluate levels of miR-181a (**A**) and ATG5 mRNA (**B**), respectively. ATG5 protein abundance (**C**) was determined by Western blotting. As demonstrated, perinatal nicotine treatment significantly enhanced expression level of miR-181-a (**A**) but attenuated both mRNA and protein levels of ATG5 (**B**,**C**) in the neonatal rat brains. Data are means ± SEM. * *p* < 0.05, control vs. nicotine. *n* = 4–6 animals/group.

Rat pup brains were isolated on postnatal day 9. qRT-PCR and Western blotting analysis were employed to evaluate miR-181a and ATG5 levels in neonatal rat brain in the presence or absence of nicotine exposure, respectively. As shown in Figure 1A, perinatal nicotine exposure significantly enhanced the expression levels of miR-181a (*p* = 0.018) in neonatal brains as compared to the controls. However, in contrast to the increased miR-181a, both mRNA (Figure 1B) (*p* = 0.0008) and protein (Figure 1C) (*p* = 0.0085) levels of ATG5 in neonatal brains were significantly attenuated in nicotine exposed group as compared with the controls. These results suggest that perinatal nicotine exposure induces an aberrant miR-181a/ATG5 signaling in developing brains.

### 2.2. miR-181a Mimic Inhibited ATG5 Protein Expression in Neonatal Rat Brain and Exaggerated H/I-Induced Brain Injury

**Figure 2:**
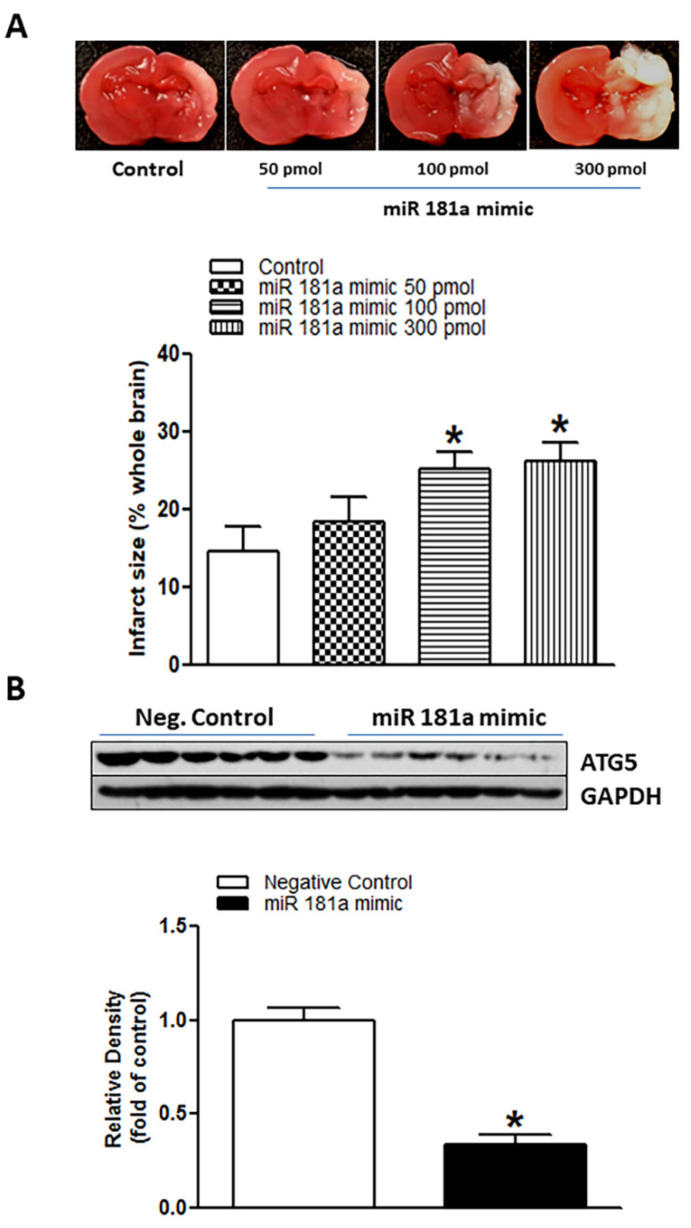
MiR-181a mimic reduced ATG5 protein level and exaggerated hypoxia/ischemia (H/I)-induced brain injury in neonatal pups. Different concentrations of miR-181a mimic and negative control were administered intracerebroventricularly (i.c.v.) on postnatal day 7 (P7) pups. Then, H/I procedure was conducted on postnatal day 9 (P9). After 48 h of H/I procedure, brain infarct size was determined by 2,3,5-triphenyltetrazolium chloride (TTC) solution staining (**A**). As shown in (**A**), miR-181a mimic administration significantly increased H/I-induced brain infarct size in a dose-dependent manner and reduced protein levels of ATG5 (**B**) in neonatal rat brains. Data are means ± SEM. * *p* < 0.05, control vs. miR-181a mimic, *n* = 7–12 animals/group. ATG5 protein level was evaluated by Western blotting in the P9 neonatal brain (**B**). Data are means ± SEM. * *p* < 0.05, control vs. miR-181a mimic (100 pmol), *n* = 6 animals/group.

In our present study, we investigated the potential roles of miR-181a in the setting of neonatal HIE. As shown in Figure 2A, miR-181a mimic intracerebroventricularly (i.c.v.) administration induced concentration-dependent increases in H/I-induced brain infarct sizes in neonatal pups, especially at moderate and high doses of 100 pmol (25.06% ± 2.295%) and 300 pmol (26.05% ± 2.558%). In addition to increase brain infarct sizes, treatment with miR-181a mimic (100 pmol) significantly decreased the levels of ATG5 protein in the neonatal brain (Figure 2B) (*p* < 0.0001). These data suggest that activation of miR-181a can directly regulate ATG5 expression and worsen H/I brain injury.

### 2.3. Inhibition of miR-181a Reversed Perinatal Nicotine Exposure-Mediated Increase in H/I-Induced Brain Injury and Eliminated the Differences of miR-181a/ATG5 Levels between Both Nicotine-Exposed and Saline Controls

**Figure 3:**
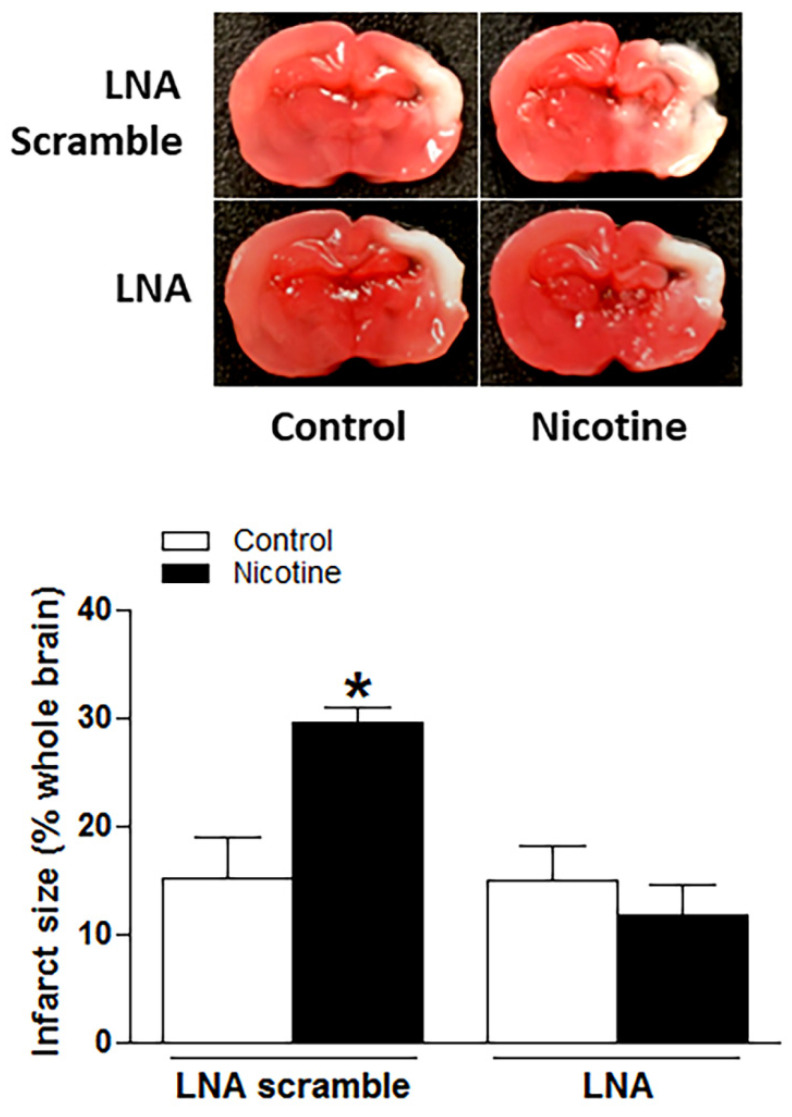
Inhibition of miR-181a reversed nicotine exposure-mediated increases in H/I-induced brain injury in neonatal offspring. Locked nucleic acid (LNA)-MiR-181a antisense and LNA scramble were administered intracerebroventricularly (i.c.v.) on P7 offspring in both saline and nicotine exposed groups. H/I procedure was conducted on P9 offspring. Brain infarct size was determined by TTC staining 48 h after H/I procedure. As presented in this figure, nicotine exposure significantly increased H/I-induced brain infarct size in LNA scramble treated animals while inhibition of miR-181a via its LNA abolished nicotine exposure-mediated enhanced infarct size in neonatal rat brain. Data are means ± SEM. * *p* < 0.05, control vs. nicotine. *n* = 8–12 animals/group.

**Figure 4:**
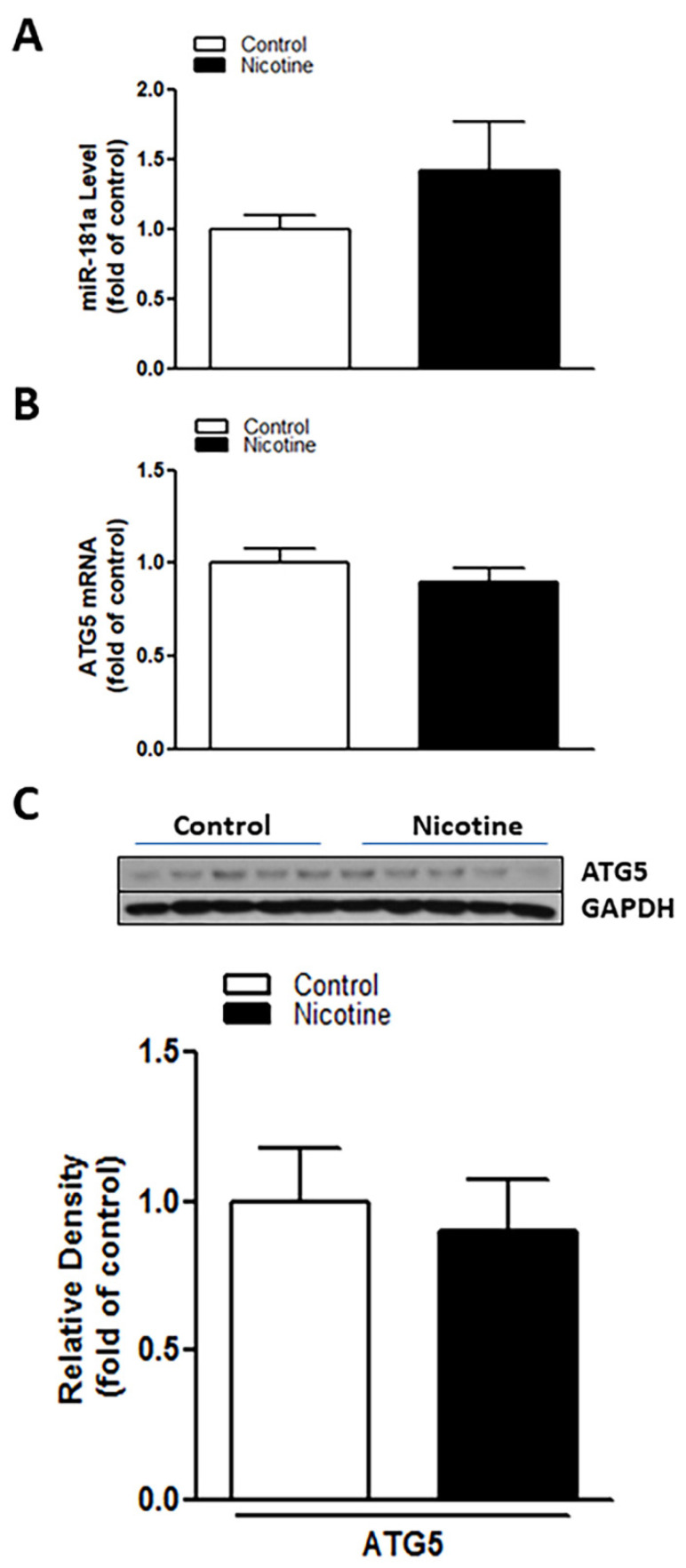
Inhibition of miR-181a recovered nicotine induced aberrant miR-181a/ATG5 levels in neonatal rat brain. LNA-MiR-181a was administered intracerebroventricularly (i.c.v.) on P7 pups. Brain was isolated on P9. qRT-PCR analysis was employed to evaluate levels of miR-181a (**A**) and ATG5 mRNA (**B**), respectively. ATG5 protein abundance (**C**) was determined by Western blotting. As shown in this figure, LNA-miR-181a administration decreased miR-181a expression and eliminated the differences of both protein and mRNA levels of ATG5 in neonatal brains between the nicotine exposed and saline control groups. Data are means ± SEM. *n* = 5 animals/group.

To determine whether the nicotine exposure-induced miR-181a overexpression plays a causal role in perinatal nicotine-mediated enhanced susceptibility to neonatal HIE in offspring, LNA-miR-181a (miR-181a antisense) was employed via i.c.v. to neonatal rat pups before H/I procedure. As shown in Figure 3, perinatal nicotine exposure enhanced H/I-induced brain infarct sizes in neonatal pups in the absence of LNA-miR-181a treatment (15.60% ± 3.542% versus 30.08% ± 1.528%). However, pre-treatment of LNA-miR-181a eliminated the difference of H/I-induced brain infarct sizes between the saline control and nicotine exposed groups (15.32% ± 2.932% versus 11.92% ± 2.421%). Furthermore, treatment with LNA-miR-181a eliminated the differences of miR-181a expression levels (Figure 4A) and also abolished the differences of both mRNA levels (Figure 4B) and protein levels (Figure 4C) of ATG5 in neonatal brain between nicotine exposed and saline control groups. These data demonstrate that miR-181a overexpression plays a causal role in nicotine exposure-mediated enhanced H/I brain injury and aberrant ATG5 signaling.

### 2.4. Perinatal Nicotine Exposure Suppressed lncRNA H19 Expression and Inhibition of H19 Enhanced miR-181a Expression Leading to an Increase in H/I-Induced Brain Injury

**Figure 5:**
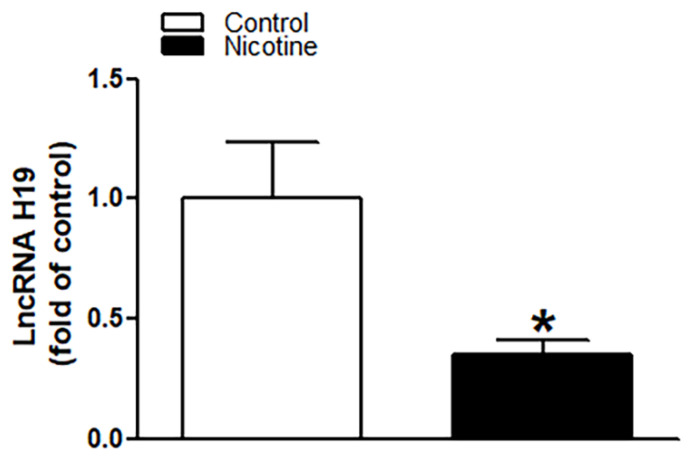
Perinatal nicotine exposure significantly attenuated the expression levels of lncRNA H19 in neonatal brain. Brains were isolated from 9-day-old neonatal rat pups in both saline control and nicotine exposed groups. qRT-PCR analysis was employed to evaluate the level of lncRNA H19 in the brain. As presented, expression levels of lncRNA H19 significantly decreased in nicotine exposed animals as compared to the saline controls. Data are means ± SEM. * *p* < 0.05, control vs. nicotine. *n* = 5 animals/group.

**Figure 6:**
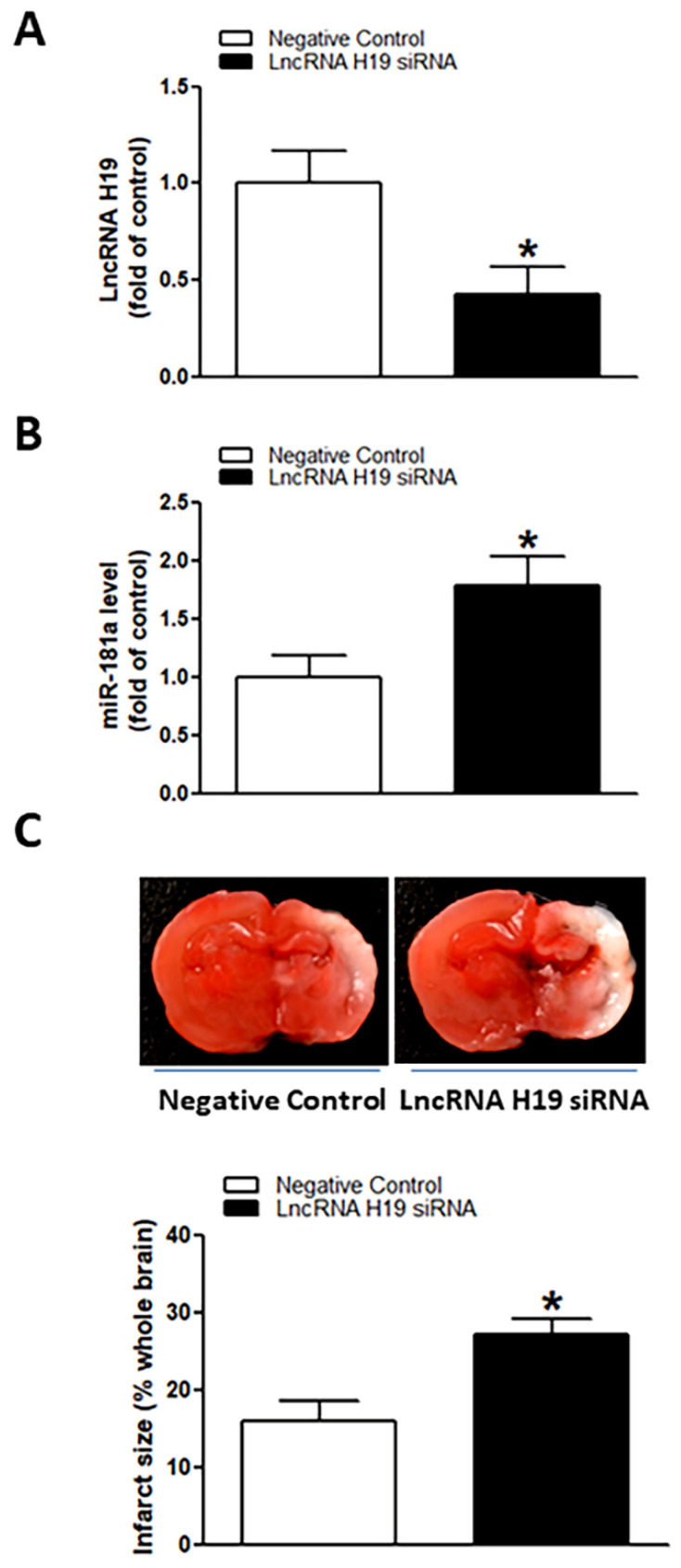
Inhibition of lncRNA H19 upregulated miR-181a expression and enhanced H/I-induced brain injury in neonatal pups. lncRNA H19 siRNA and negative control were administered intracerebroventricularly (i.c.v.) on postnatal day 7 (P7) pups, and H/I procedure was conducted on P9 pups. Levels of lncRNA H19 (**A**) and miR-181a (**B**) were evaluated by qRT-PCR analysis in neonatal rat brain on P9 pups. LncRNA H19 siRNA administration not only reduced expression levels of lncRNA H19 but also significantly enhanced expression of miR-181a in neonatal rat brains (**A**,**B**). Of importance, knockdown of lncRNA H19 also increased H/I-induced brain infarct size as compared to the negative control group (**C**). Data are means ± SEM. * *p* < 0.05, control vs. lncRNA H19 siRNA. *n* = 5 animals/group. Brain infarct size (**C**) was determined by TTC staining 48 h after H/I procedure. Data are means ± SEM. * *p* < 0.05, control vs. lncRNA H19 siRNA. *n* = 9–11 animals/group.

As shown in Figure 5, perinatal nicotine exposure significantly attenuated the expression levels of lncRNA H19 in neonatal rat brain as compared with the controls. We further evaluated whether there is a potential association between nicotine-mediated lncRNA H19 repression and miR-181a overexpression in neonatal brains. As shown in Figure 6, treatment with lncRNA H19 siRNA significantly not only inhibited the expression levels of H19 (Figure 6A) but also increased expression of miR-181a (Figure 6B) in neonatal rat brains. It is important that knockdown of lncRNA H19 with siRNA also significantly enhanced H/I-induced brain injury in neonates (15.98% ± 2.628% versus 27.06% ± 2.149%) (Figure 6C). These data indicate that lncRNA H19 serves as one upstream negative regulator in nicotine exposure-mediated aberrant miR-181a signaling and enhanced H/I brain injury.

## 3. Discussion

Emerging evidence reveals the critical roles of non-coding RNAs in brain development and pathologies. Our present study further enriched this notion. There are several novel findings obtained in the present study. Firstly, we revealed that perinatal nicotine exposure enhanced miR-181a in the neonatal brain while enhancement of miR-181a activity with its mimic suppressed ATG5 expression and increased H/I-induced brain infarct size in neonatal pups. Secondly, we further verified that inhibition of miR-181a with LNA-miR-181a antisense reduced perinatal nicotine-mediated miR-181a overexpression and eliminated the differences of ATG5 expression and H/I-induced brain infarct size between nicotine exposed and saline control groups. Lastly, we also found that perinatal nicotine exposure attenuated LncRNA H19 expression and inhibition of H19 resulted in upregulation of miR-181a expression and enhanced H/I-induced brain injury in neonates. These findings suggest that alteration of H19/miR-181a/ATG5 signaling plays a key role in the fetal programming of neonatal hypoxia/ischemia-sensitive phenotype of the brain in response to maternal nicotine use.

Large amounts of studies suggest a pivotal role of miR-181a in the pathogenesis of cardiovascular and neurological diseases [14,15,16,17,30]. They have shown that miR-181a has dual effects in the setting of brain ischemic disease including detrimental and protective effects; however, most previous studies have shown a dominant detrimental effect of miR-181a over-expression in various brain pathologies such as ischemic brain injury, traumatic brain injury, and epilepsy, while antagonizing excessive miR-181a activity confers neuroprotective effects [18,19,20,21,22,38,39]. Xu et al. reported that treatment with miR-181a antagomir reduces injury and improves long-term behavioral recovery in a mice model of focal cerebral ischemia [22]. Consistent with these observations, our present findings that enhancement of miR-181a expression with miR-181a mimic in the neonatal pups significantly increased H/I-induced brain infarct size further suggest a detrimental effect of miR-181a in the pathology of neonatal HIE.

As one of the pivotal epigenetic mechanisms, miRNAs are sensitive to various perinatal stressful insults, such as maternal undernutrition, smoking exposure, restraint of the body and forced swimming, etc., resulting in aberrant regulation of target gene expression in the developing fetus and contributing to the development of diverse neurological diseases in offspring [40,41,42]. Zucchi et al. has demonstrated that prenatal stresses (body restraint and forced swimming test) modify epigenetic signatures through a miRNA signaling pathway linked to disease during critical periods of fetal brain development [42]. In addition, previous studies [41] have shown that maternal cigarette smoking during pregnancy has been associated with altered expression of miRNAs, but how these changes of miRNAs may affect later offspring health outcomes remain undetermined. Growing evidence has indicated the vital roles of miR-181a in the pathogenesis of cardiovascular and neurological diseases [14,15,16,17,30]. Several studies have also demonstrated that overexpression of miR-181a confers potential detrimental effects in various brain pathologies [18,19,20,21,22]. In agreement with these studies, our present study also demonstrated that perinatal nicotine exposure caused a developmental upregulation of miR-181a expression in the neonatal brain. Of importance, our current findings that inhibition of miR-181a via LNA-miR-181a antisense eliminated the differences of brain infarct size in neonatal HIE between nicotine exposed and saline control groups suggest that the over-expressed miR-181a plays a causal role in perinatal nicotine exposure-meditated development of brain ischemia-sensitive phenotype. Therapeutic targeting miR-181a could prevent or reverse perinatal nicotine exposure-induced adverse pathological effects on brain in offspring. In previous studies, we observed that perinatal nicotine exposure selectively enhanced miR181a expression in vascular tissues, which directly upregulated coronary vascular tone leading to the enhanced heart dysfunction and heart ischemic injury [30]. These findings suggest that aberrant over-expression of miR-181a serves as one of the common pathways in maternal nicotine exposure-induced adverse developmental disease in offspring and antagonizing excess of miR-181a may be an effective therapeutic target to rescue perinatal nicotine-mediated brain and cardiovascular disorders in offspring.

Autophagy is a well-documented classic mechanism that ensures the maintenance of homeostasis in response to diverse intracellular and extracellular stresses [43,44,45,46]. Accumulating evidence indicates that defective basal autophagic responses play a vital role in the pathogenesis of acute ischemic brain insults [47,48,49,50]. Our recent study revealed that basal level of autophagy activity plays an essential role in neonatal rat brain while disruption of such basal autophagic flux exacerbates brain ischemic injury [51]. Knockdown of AGT5, a key component of autophagic flux, significantly enhanced hypoxia/ischemia-induced neonatal brain injury [52]. Emerging evidence suggests a potential linkage between miR-181a and autophagy activation. It has shown that miRNA-181a impairs physiological and pathological processes via target autophagy-related genes [53,54,55,56]. Our present findings that miR-181a mimic enhanced hypoxia/ischemia-induced brain injury associated with a decrease in ATG5 expression, suggest ATG5 is one of the targets of miR-181a and an important molecular linker between miR-181a and brain ischemic injury. Indeed, previous studies have suggested that miR-181a is a novel and important regulator of autophagy and ATG5 is a rate-limiting miR-181a target in this effect [55,56]. Our present study shows that perinatal nicotine exposure attenuated ATG5 gene expression and abundance in offspring neonatal brains. Furthermore, inhibition of miR-181a effectively normalized the repressed ATG5 gene in the nicotine exposed neonatal rat brain as compared with the saline controls. These findings clearly imply that ATG5 is one of the direct downstream targets of miR-181a in neonatal brains. From these findings, we can speculate that perinatal nicotine exposure-induced miR-181a overexpression directly targets the ATG5 gene and inhibits ATG5 expression leading to autophagy deficiency. This autophagy deficiency could lose its capacity to clear the hypoxia/ischemia-induced brain injured cells and delay brain recovery after neonatal Hypoxia/ischemia stimulation.

The present findings that perinatal nicotine exposure attenuated lncRNA H19 expression levels in neonatal brains as compared with the saline controls suggest that H19 may be one of the key regulatory factors in perinatal nicotine exposure-mediated brain ischemic pathology. In comparison to other small non-coding RNAs, lncRNAs are abundant RNA transcripts of more than 200 nucleotides, transcribed by RNA polymerase II from different regions of a genome [57,58]. LncRNAs can be present in the cytoplasm and/or nucleus and act as an epigenetic factor or sponger to regulate gene expression by binding to miRNA, mRNA, or DNA [57,58]. H19 is the first identified lncRNA, which plays an important role in regulation of cell homeostasis and pathological processes [31,32,34,59,60,61]. Recent studies suggest potential interactions between lncRNA H19 and miR-181a [33,35]. In agreement with these findings, our current study demonstrated that knockdown of H19 with an siRNA approach in neonatal brains significantly enhanced hypoxia/ischemia-induced brain injury associated with an increase in miR-181a expression. These findings validate that H19 is an upstream negative regulator of miR-181a signaling. The perinatal nicotine exposure-mediated H19 repression may contribute to the miR-181a overexpression and the development of brain ischemia-sensitive phenotype in postnatal offspring.

## 4. Materials and Methods

### 4.1. Experimental Animals and Nicotine Treatment

Pregnant Sprague–Dawley rats were purchased from Charles River Laboratories (Portage, MI, USA) and were randomly divided into 2 groups: (1) saline control; and (2) nicotine treatment group. Nicotine or saline solution was administrated via osmotic minipumps implanted subcutaneously from day 4 of gestation to day 10 after birth as we described previously [12,13]. Briefly, pregnant rats were anesthetized with isoflurane, an incision was made on the back, and osmotic minipumps (type 2ML4; Alza, Palo Alto, CA, USA) were implanted subcutaneously. The incision was closed with four or five sutures. About half of the pregnant rats were implanted with the minipumps containing nicotine at a concentration of 102 mg/mL, and the other half were implanted with the minipumps containing only saline, which served as the vehicle control. The flow rate of the minipumps was 60 μL/day, which delivered a dose of 2.1 mg of nicotine free-base per day. In rats of an average of 350 g bodyweight, this corresponds to a dose rate of 6 mg/kg/day, which closely mimics the doses occurring in moderate to heavy human smokers [62,63]. According to the manufacturer’s specifications, the delivery period for the pumps is 28 days, and thus delivery continued after birth until postnatal day 10. Totally, 34 pregnant rats of 3-month-old age with body weight of 204 ± 20 g were used for this study. Nicotine treatment did not affect the litter size and the length of gestation, and all these pregnancies reached full term. Offspring studies were conducted on P9 male pups—4–6 pups/group for molecular studies such as Western blotting and qRT-PCR, and 10–15 pups/group for HIE functional study. To eliminate the litter effects, pups for each group were from different dams. All procedures and protocols (IACUC# 20146, approved on 9 September 2020) were approved by the Institutional Animal Care and Use Committee of Loma Linda University and followed the guidelines by the National Institutes of Health Guide for the Care and Use of Laboratory Animals.

### 4.2. Neonatal Hypoxic Ischemic Encephalopathy (HIE) Model and Infarct Size Measurement

An HIE procedure was performed in P9 pups using a modified Rice–Vannucci model [64]. Briefly, the pup was placed on a surgical table maintained at 37 °C and fully anesthetized with inhalation of isoflurane (3–4% for induction and 2% for maintenance). A small incision was made in the neck to expose the right common carotid artery (CCA). The CCA was double ligated with a 5.0 silk surgical suture and then cut in the middle of two ligation sites. The pups were returned to their dam to recuperate for about 1 h. Pups were then placed in a hypoxic incubator (Isotemp, Fisher Scientific Company, Hampton, NH, USA) which contains humidified 8% oxygen balanced with 92% nitrogen for 2 h at 37 °C. At the end of hypoxia, pups were returned to their dam for recovery. For sham group, the pups had the same surgery procedure performed only without ligation of their right common carotid arteries and without hypoxia exposure.

To determine the HIE injury, the brain infarct size will be measured by triphenyltetrazolium chloride (TTC) staining. Briefly, the pups were deeply anesthetized with 5% isoflurane and then euthanized by decapitation at 48 h after the HIE treatment. Coronal slices of the brain (2 mm thick) were cut and immersed into a 2% TTC solution for 5 min at 37 °C and then fixed by 10% formaldehyde overnight. Each slice was photographed separately, and the percentage of infarction area for each slice was analyzed by ImageJ software (Version 1.40; National Institutes of Health, Bethesda, MD, USA), summed for each brain, and expressed as a percentage of whole brain.

### 4.3. Intracerebroventricular Injection (i.c.v.)

miR-181a antisense (LNA-miR-181a,100 pmol), miR-181a mimic (50, 100, 300 pmol), lncRNA H19 siRNA (100 pmol), and their corresponding negative controls were prepared as manual instructions and administered intracerebroventricularly 48 h before the HI treatment, respectively. As previously described [12,13], the pups were anesthetized and fixed on a stereotaxic apparatus (Stoelting, Wood Dale, IL, USA). An incision was made on the skull surface and bregma was exposed. All agents were injected at a rate of 1 μL/min, a total volume of 2 μL, with a 10-L syringe (Stoelting) on the right hemisphere following the coordinates relative to bregma: 2 mm posterior, 1.5 mm lateral, and 3.0 mm below the skull surface. The injection lasted 2 min, and the needle was kept for an additional 10 min before its removal. The incision was sutured.

### 4.4. Western Blotting Analysis

Brain samples were isolated from P9 rat pups and were homogenized with a lysis buffer containing 150 mmol/L NaCl, 50 mmol/L Tris HCl, 10 mmol/L EDTA, 0.1% Tween 20, 1% Triton, 0.1% β-mercaptoethanol, 0.1 mmol/L phenylmethylsulfonyl fluoride, 5 μg/mL leupeptin, and 5 μg/mL aprotinin, pH 7.4. Homogenates were centrifuged at 4 °C for 15 min at 14,000× *g*. The supernatants were then collected and protein concentrations were determined via a protein assay kit (Bio-Rad, Hercules, CA, USA). Samples with equal amounts of protein (30 μg) were loaded onto 10% polyacrylamide gel with 0.1% sodium dodecyl sulfate and separated by electrophoresis at 100 V for 90 to 120 min. Proteins were then transferred onto nitrocellulose membranes. Nonspecific binding sites were blocked for 3 to 4 h at room temperature in a Tris-buffered saline solution containing 5% dry milk. The membranes were probed with primary antibodies against ATG5 (1:1000, cell-signaling), as described previously [12,13]. After washing, membranes were incubated with secondary horseradish peroxidase conjugated antibodies. Proteins were visualized with enhanced chemiluminescence reagents, and blots were exposed to Hyperfilm. The results were analyzed with Kodak ID image analysis software. Band intensities were normalized to glyceraldehyde-3-phosphate dehydrogenase.

### 4.5. Real-Time qRT-PCR for miR-181a Quantification

MiR-181a levels were determined by miScript II RT kit (Qiagen Company, Hilden, Germany) and miScript SYBR Green PCR kit with miScript Primer Assay kit (Qiagen) according to manufacturer’s instructions. Primers included miScript universal primer, miR-181a miScript primer assay (Rn_miR-181a_2; Cat#MS00013125; Qiagen) and SNORD61 miScript primer assay (Hs_SNORD61_11; Cat#MS00033705; Qiagen). Briefly, 2 μg of template RNA were mixed with a reverse-transcription master mix in a final volume of 20 μL and incubated for 60 min at 37 °C, and the reaction was stopped at 95 °C. Two nanograms of template cDNA were used for miR-181a quantification in a final volume of 25 μL system containing specific primers and QuantiTect SYBR Green PCR master mix following manufacturer’s instructions. Serial dilutions of the positive control were done on each plate to create a standard curve for the quantification. PCR was done in triplicate and the threshold cycle numbers were averaged for each sample. The relative miR-181a levels were calculated as the Ct values of miR-181a to the reference gene (SNORD61).

### 4.6. Real-Time qRT-PCR for ATG5 mRNA and lncRNA H19 Quantification

Briefly, total RNA was extracted using the TRIzol reagent (Invitrogen, Waltham, MA, USA) and subjected to reverse transcription with All-in-One cDNA Synthesis SuperMix (B24403, Bimake, Houston, TX, USA), following the manufacturer’s instructions. The ATG5 mRNA and lncRNA H19 abundance were determined with real-time PCR using iQ SYBR Green Supermix (Bio-Rad). Primers included: ATG5, forward: CTCTGCCTTGGAACATCACA; reverse: ATCATTCTGCAGTCCCATCC. RT lncRNA qPCR Assay for Rat H19 was obtained from Qiagen (LPR07420A-200; Qiagen). Real-time PCR was performed in a final volume of 25 μL, and each PCR reaction mixture consisted of specific primers and iQ SYBR Green Supermix. Serial dilutions of the positive control were done on each plate to create a standard curve for the quantification. PCR was done in triplicate and the threshold cycle numbers were averaged for each sample. The relative ATG5 mRNA level and lncRNA H19 level were calculated as the Ct values of respective primer to the reference gene (GAPDH).

### 4.7. Statistical Analysis

All statistical analysis was performed using GraphPad Prism 5 (GraphPad software). Data are expressed as mean ± SEM. Experimental number (n) represents neonates from different dams. Statistical significance (*p* < 0.05) was determined by analysis of variance followed by Neuman–Keuls post hoc testing or Student *t*-test, where appropriate.

## 5. Conclusions

Our present study demonstrated for the first time that perinatal nicotine exposure induces an aberrant over-expression of miR-181a in the neonatal rat brain, which further targets ATG5 and contributes to the development of a sensitive phenotype to neonatal HIE, while lncRNA H19 acts as an upstream regulator of miR-181a. Importantly, inhibition of miR-181a can rescue nicotine-mediated brain damage in offspring. These findings provide a novel mechanism that aberrant lncRNA H19/miR-181a/AGT5 axis may play a vital role in perinatal nicotine exposure-mediated ischemia-sensitive phenotype in offspring and also confer promising molecular targets for intervention and rescuing nicotine-induced adverse programming effects in offspring. Given the high mortality and morbidity of neonatal HIE as well as the extensive abuse of nicotine products during pregnancy currently, it is highly warranted to further explore the potential epigenetic mechanisms underlying the perinatal stresses-induced brain developmental defects in postnatal life.

## Data Availability

The data presented in this study are available in article.
